# Prenatal cerebrospinal fluid modulate differentiation and proliferation of rat pheochoromocytoma PC12 cells

**DOI:** 10.1186/1743-8454-7-S1-S34

**Published:** 2010-12-15

**Authors:** Mohammad Nabiuni, Javad Rasouli, Kazem Parivar, Homa Mohseni Kochesfhani

**Affiliations:** 1 Department of Biology Cell and Developmental Research Lab, Tarbiat Moallem University, Tehran, Iran

## Background

During the early stages of brain development, ependymal cells that line the neural tube are thought to secrete CSF. It is well documented that fetal CSF contains many neurotrophic and growth factors which are known as modulators of neurogenesis, differentiation and brain extracellular microenvironment. Rat pheochromocytoma PC12 cells have been widely used as an in vitro model of neuronal differentiation since the cells undergo differentiation to sympathetic neuron-like cells in response to NGF, bFGF, EGF, TGF-α and GDNF. We hypothesized that prenatal CSF could have differentiational effect on PC12 cells.

## Materials and methods

CSF was removed by tapping the cisterna magna of Wistar rat fetuses (E17- E20) then centrifuged at 4000 r.p.m for 10 min, the supernatant frozen immediately and stored at -70°C until used. PC12 cells were cultured in RPMI-1640 with 10% FBS, 100 unit/ml of penicillin, 100 mg/ml of streptomycin and 5% CO2 at 37°C. For culture experiments, 2*10E4 PC12 cells were added to each well of a 96-well plate that had been coated with Poly-D-Lysine. After attachment, the cells were exposed to CSF at different ages with dissimilar concentration of 7, 10, 25% (v/v). The cell viability and cell proliferation were measured by MTT assay. The neuronal differentiation of PC12 cells were considered by changes of neurite outgrowth.

## Results

Viability and cell proliferation were significantly elevated in PC12 cells cultured in CSF supplemented medium in E18 compared with control ones. A significant neuronal-like outgrowth appeared as early as Day 3 after the application of the CSF supplemented medium E17.

## Conclusions

It was shown that CSF neurotrophic factors can support normal neurogenesis and promotes proper brain development, neuronal differentiation. It has been reported that CSF can be a survival material on its own with any medium for cerebral cortex primary cultures. Our data are in the same line with pervious studies that clarify crucial role of CSF neurotrophic factors in neuronal differentiation and cell proliferation. Taken together we address PC12 neuronal differentiation and cell proliferation to CSF induction by its components especially growth factors.

